# Bone Quality Assessment Before Total Hip Arthroplasty: The Role of Densitometry

**DOI:** 10.7759/cureus.55480

**Published:** 2024-03-04

**Authors:** Iga Żarnowska, Bartłomiej Wilk, Milena Chilińska, Kamil Kołodziejczyk, Rafał Garlewicz, Marcin Zlotorowicz

**Affiliations:** 1 Department of Internal Medicine, Warsaw Southern Hospital, Warsaw, POL; 2 Department of Orthopedics and Traumatology, Medical University of Warsaw, Warsaw, POL; 3 Department of Spine Disorders and Orthopaedics, Gruca Teaching Hospital, Otwock, POL; 4 Department of Orthopedics, Pediatric Orthopedics, and Traumatology, Gruca Teaching Hospital, Otwock, POL

**Keywords:** osteoarthritis, preoperative planning, tha, total hip arthroplasty, densitometry

## Abstract

Background

Total hip arthroplasty (THA) is effective in the treatment of hip osteoarthritis. Radiographic evaluation, standard in THA planning, is sufficient in examining hip anatomy, although it may not precisely assess bone quality. A routinely implemented method in bone quality assessment is densitometry. The technique allows for a measurement of bone mineral density (BMD).

Methodology

In the study, we included 26 participants who qualified for THA. All the patients were preoperatively examined with radiographs and densitometry of the affected hip. On the preoperative anteroposterior radiograph, we measured the canal-to-calcar isthmus ratio (CC ratio) and the cortical index (CI). Intraoperatively, during the THA procedure, we measured the thickness of the cortical bone and the diameter of the femoral neck in the line of neck resection.

Results

The examination with Pearson’s correlation coefficient revealed that BMD significantly positively correlates with the intraoperatively measured diameter of the femoral neck (*r *= 0.5, *P *= 0.009), and with the measured thickness of the cortical bone (*r *= 0.47, *P *= 0.015), CI significantly positively correlates with the intraoperatively measured diameter of the femoral neck (*r *= 0.6, *P *= 0.001), and with the CC ratio (*r *= 0.44, *P *= 0.024), the intraoperatively measured diameter of the femoral neck significantly positively correlates with the intraoperatively measured thickness of the cortical bone (*r *= 0.59, *P *= 0.001). All of the other correlations were not statistically significant.

Conclusions

BMD measurements can be used in THA planning as they positively correlate with intraoperative measurements. The radiological parameters (CC ratio and CI) may not be as precise in bone quality assessment.

## Introduction

Osteoarthritis (OA) is the most common type of arthritis affecting human joints [1]. It is estimated that approximately 7% of adults older than 25 years report the symptoms of OA [2]. The disease is a serious problem since it remarkably decreases the patient’s quality of life even though it may be underestimated by healthcare professionals [3]. Fortunately, when it comes to end-stage hip OA, we can offer help to the patients in the form of total hip arthroplasty (THA). The procedure is well proven to improve the quality of life and clinical outcomes both in the long and short term after the operation [4,5]. In addition, THA is an effective procedure, with patients reporting high postoperative satisfaction levels [6,7].

Such optimistic outcomes may be achieved thanks to proper preoperative planning and implant choice [8]. Nowadays, in THA, we can utilize various types of implants. When it comes to placement methods, we can use cementless or cemented implants. In addition, we can choose from different sizes of implant components and stem lengths to achieve optimal combinations. Finally, in some cases, we can incorporate variations from the classic method such as dual-mobility THAs. This variety of options requires the surgeon to carefully assess the preoperative setup, to plan an adequate treatment strategy.

There are several methods utilized in THA preoperative planning. The standard method is the radiographic evaluation that includes an anteroposterior (AP) radiograph of the pelvis, an AP radiograph of the hip, and an axial radiograph of the hip. In most patients, such imaging allows for a complete review [8]. In some cases, such as dysplasia of the hip, bone deficiency, or postoperative changes, implementing a computed tomography (CT) examination in the planning might be necessary since the technique allows for more accurate three-dimensional visualization of the anatomical structures. Some surgeons decide to utilize artificial intelligence in analyzing the imaging [9]. However, to our knowledge, such methods are not as common as classic radiographic evaluation.

The aforementioned methods are sufficient in examining the preoperative hip anatomy. Although concerning the bone tissue quality assessment, they might not be precise enough.

The optimal implant choice is based not only on the density of the bone in the proximal femur region but also on the relation between the thickness of the cortical bone and the cancellous bone [10,11]. A traditional radiograph does not allow to gather all of the particular information.

These additional data may seem unnecessary. Considering that, in many cases, the final implant choice is based on intraoperative assessment, we can assume that the additional data might be useful in preoperative planning [8].

A routinely implemented method in bone quality assessment is densitometry. The technique allows for a precise measurement of bone mineral density (BMD). The quantified data, presenting the BMD of the proximal femur, acquired priorly to THA may help the surgeon to choose an optimal implant for the patient.

Based on our experience, cases where the surgeon has to change the previously planned implant intraoperatively due to the discrepancy between the preoperative and intraoperative bone quality assessment are not uncommon. To avoid such situations we decided to examine several techniques that give information about the bone quality in the proximal femur region.

The study aims to compare the methods of bone quality assessment in the proximal femur region: BMD measurements acquired with densitometry, radiological findings, and intraoperative findings during THA.

## Materials and methods

The study was conducted in the Department of Orthopedics, Pediatric Orthopedics, and Traumatology in the Gruca Teaching Hospital, Otwock, Poland, in the years 2022 and 2023. The participants were the patients qualified for the THA procedure. In the study, we included a group of 26 participants - 10 women and 16 men. The age of the patients was between 27 and 78 years. Before the operation, all the patients were examined with an AP radiograph of the pelvis, AP and axial radiographs of the hips, and the densitometry of the affected hip with the dual-energy X-ray absorptiometry (DXA) method. The DXA examination was performed in the horizontal position, with patients lying on their backs. The DXA scanner was calibrated every day before the measurements with the quality assurance block. All the patients qualified for the procedure were diagnosed with hip joint OA, and none of the patients were diagnosed with osteoporosis. We ruled out osteoporosis by assessing the T-score of the patients. Patients with a T-score below -2.5 were excluded from the study. The average T-score of the patients was -0.16 (standard deviation [SD] = 1.06), the minimal value noted was -1.6 and the maximal value noted was 3.8.

Additionally, to assess the relations between the cortical and the cancellous bone, on the preoperative AP radiograph of the affected hip, we measured the following:

- The canal-to-calcar isthmus ratio (CC ratio): To obtain this measurement, the reference line was drawn throughout the apex of the lesser trochanter. Next, we drew a line 3 cm below the reference line. On this line, we identified two points of the endosteal margin of the femur. Finally, we drew a line 10 cm below the reference line. On this line, we also identified two points of the endosteal margin of the femur. All the lines mentioned above were drawn perpendicularly to the long axis of the femur. Next, we marked the lateral and the medial endosteal lines by connecting the previously identified lateral and medial points of the endosteal margin. After doing so we identified the distance (X) defined as the distance between two points of the endosteal margin of the femur on the line 10 cm below the reference line and the distance (Y) defined as the distance between points where the lines created by connecting the lateral and the medial points of the endosteal margin cross the reference line. The CC ratio was calculated by dividing the distance (X) by the distance (Y).

- The cortical index (CI): To obtain this measurement, the reference line was drawn throughout the apex of the lesser trochanter. Next, we drew a line 10 cm below the reference line. On this line, we identified the distance (X) between two points of the endosteal margin of the femur and the distance (Z) between two periosteal surfaces. All the lines were drawn perpendicularly to the long axis of the femur. The CI was calculated by dividing the difference between the distance (Z) and the distance (X) by the distance (Z).

**Figure 1:**
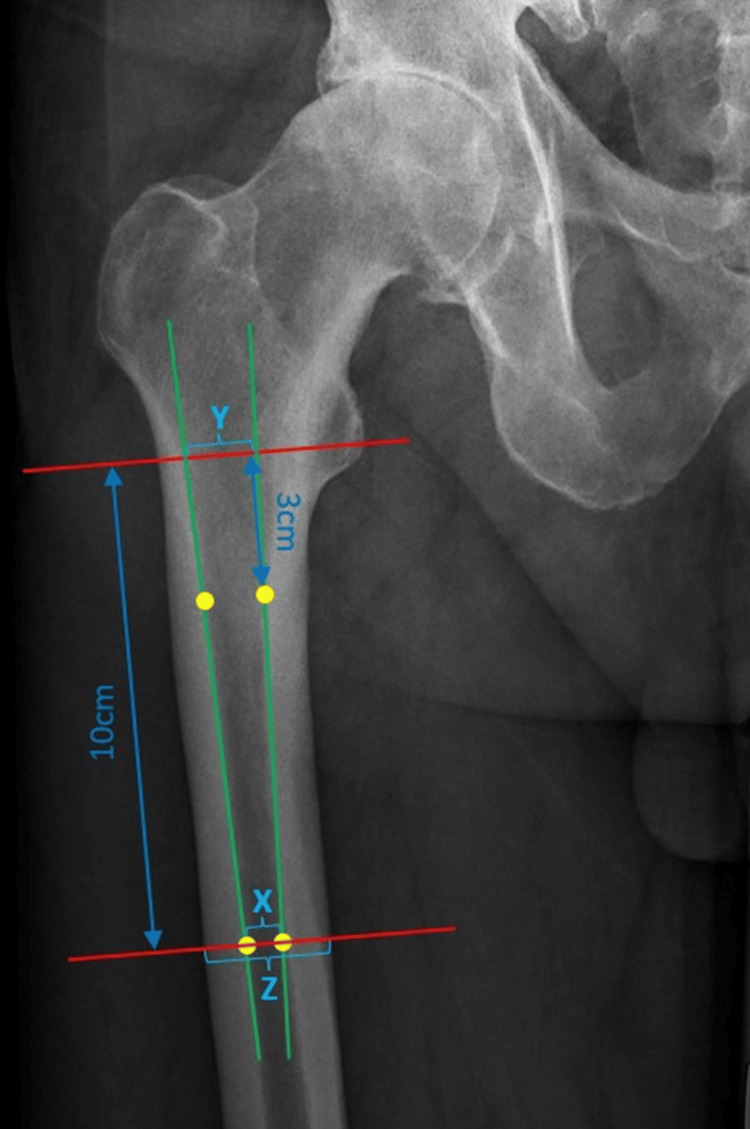
Representation of the radiological measurements taken in the study. All the measurements were made according to the method previously described by Dorr et al. [10]. In the preoperative AP radiograph of the affected hip, we identified three lines perpendicular to the long axis of the femur: the reference line crossing the apex of the lesser trochanter, the line 3 cm below the reference line, and the line 10 cm below the reference line. On both the line 3 cm and the line 10 cm below the reference line, we identified two points along the endosteal margin of the femur. By connecting the lateral and the medial points of the endosteal margin we identified the lateral and the medial endosteal lines. Next, we identified three distances: the distance (X) between two points of the endosteal margin of the femur on the line 10 cm below the reference line, the distance (Y) between the points where the lateral and the medial endosteal lines cross the reference line and the distance (Z) between two periosteal surfaces on the line 10 cm below the reference line. CC ratio was calculated by dividing the distance (X) by the distance (Y). CI was calculated by dividing the difference between the distance (Z) and the distance (X) by the distance (Z). CI, cortical index; CC, canal-to-calcar isthmus

All the measurements were made with the method previously described by Dorr et al. [12]. We depicted our measurements in Figure 1.

The intraoperative measurements were made during the THA procedure. We measured the thickness of the cortical bone and the diameter of the femoral neck in the line of neck resection standard for the implant with the long or medium-length stem. The measurements were made in millimeters using a caliper.

All the numerical data were collected and stored in the anonymized form in Microsoft Excel. The data supporting the findings of this study can be provided by the corresponding author upon reasonable request.

Statistical analysis of the data was performed using Statistica Version 13.3 for Windows (TIBCO Software Inc., Palo Alto, CA). The normal distribution of the parameters was checked using the Shapiro-Wilk test. After proving normal data distribution, we used Pearson’s correlation coefficient to examine the relationship between the variables.

The procedures performed were in accordance with the ethical standards of the Institutional Review Board (IRB) as well as with the 1964 Helsinki Declaration and its later amendments. Informed consent was obtained from all individual participants included in the study. The study was approved by the Bioethics Committee of the Center of Postgraduate Medical Education in Warsaw, Poland (May 12, 2021/No. 41/2021).

## Results

We analyzed the relations between the age of the patients, CC ratio, Cl, BMD, intraoperatively measured diameter of the femoral neck in the line of neck resection, and intraoperatively measured thickness of the cortical bone in the line of neck resection. As mentioned earlier, we used Pearson’s correlation coefficient to examine the relations minding normal data distribution.

The overview of the most important baseline characteristics of data is given in Table 1.

**Table 1:** Baseline characteristics. In the table, we included the details of the most important parameters measured in our study to give a better insight into the data. We listed the average value, median, standard deviation, minimal value, maximal value, and the number of observations of the following parameters: the age of the patients, intraoperatively measured diameter of the femoral neck in the line of neck resection, the intraoperatively measured thickness of the cortical bone in the line of neck resection, and BMD. BMD, bone mineral density

Baseline characteristics	Age (years)	Intraoperatively measured diameter of the femoral neck in the line of neck resection (mm)	Intraoperatively measured thickness of the cortical bone in the line of neck resection (mm)	BMD (g/cm^2^)
Average	63.58	39.73	5.04	0.98
Median	64.00	40.00	5.00	0.98
Standard deviation	10.67	4.29	2.07	0.18
Minimum	27.00	31.00	2.00	0.59
Maximum	78.00	49.00	10.00	1.34
Number of observations	26.00	26.00	26.00	26.00

Examination of the relations between the parameters revealed the following: -BMD significantly positively correlates with the intraoperatively measured diameter of the femoral neck in the line of neck resection (*r *= 0.5, *P *= 0.009). -BMD significantly positively correlates with the intraoperatively measured thickness of the cortical bone in the line of neck resection (*r *= 0.47, *P *= 0.015). -CI significantly positively correlates with the intraoperatively measured diameter of the femoral neck in the line of neck resection (*r *= 0.6, *P *= 0.001). -The intraoperatively measured diameter of the femoral neck in the line of neck resection significantly positively correlates with the intraoperatively measured thickness of the cortical bone in the line of neck resection (*r *= 0.59, *P *= 0.001). -CI significantly positively correlates with the CC ratio (*r *= 0.44, *P *= 0.024).

The overview of the statistically significant relations between the parameters is presented in Table 2.

**Table 2:** Statistically significant correlations. In the table, we included the statistically significant correlations between the parameters measured in our study to provide a better understanding of the results. BMD, bone mineral density; CI, cortical index

Parameter 1	Parameter 2	Pearson’s correlation coefficient (*r*)	*P*-value
BMD	Intraoperatively measured diameter of the femoral neck in the line of neck resection	0.5	0.009
BMD	Intraoperatively measured thickness of the cortical bone in the line of neck resection	0.47	0.015
CI	Intraoperatively measured diameter of the femoral neck in the line of neck resection	0.6	0.001
Intraoperatively measured diameter of the femoral neck in the line of neck resection	Intraoperatively measured thickness of the cortical bone in the line of neck resection	0.59	0.001
CI	CC ratio	0.44	0.024

All of the other correlations between the age of the patients, CC ratio, Cl, BMD, intraoperatively measured diameter of the femoral neck in the line of neck resection, and intraoperatively measured thickness of the cortical bone in the line of neck resection were not statistically significant.

All the correlations and their statistical significance are listed in Table 3.

**Table 3:** Correlations. In the table, we present the correlations between all of the parameters measured in our study: the age of the patients, intraoperatively measured diameter of the femoral neck in the line of neck resection, intraoperatively measured thickness of the cortical bone in the line of neck resection, CC ratio, Cl, and BMD. The correlations were examined using Pearson’s correlation coefficient (*r*). BMD, bone mineral density; CI, cortical index; CC, canal-to-calcar isthmus

Correlations		Age (years)	Intraoperatively measured diameter of the femoral neck in the line of neck resection (mm)	Intraoperatively measured thickness of the cortical bone in the line of neck resection (mm)	CC ratio	CI	BMD (g/cm^2^)
Age (years)	Pearson’s correlation coefficient (*r*)		-0.130	-0.037	0.304	0.129	0.085
	Statistical significance (*P*)		0.527	0.856	0.132	0.531	0.679
Intraoperatively measured diameter of the femoral neck in the line of neck resection (mm)	Pearson’s correlation coefficient (*r*)	-0.130		0.591	0.192	0.597	0.502
	Statistical significance (*P*)	0.527		0.001	0.348	0.001	0.009
Intraoperatively measured thickness of the cortical bone in the line of neck resection (mm)	Pearson’s correlation coefficient (*r*)	-0.037	0.591		0.124	0.225	0.472
	Statistical significance (*P*)	0.856	0.001		0.546	0.268	0.015
CC ratio	Pearson’s correlation coefficient (*r*)	0.304	0.192	0.124		0.441	-0.255
	Statistical significance (*P*)	0.132	0.348	0.546		0.024	0.208
CI	Pearson’s correlation coefficient (r)	0.129	0.597	0.225	0.441		0.080
	Statistical significance (*P*)	0.531	0.001	0.268	0.024		0.699
BMD (g/cm^2^)	Pearson’s correlation coefficient (*r*)	0.085	0.502	0.472	-0.255	0.080	
	Statistical significance (*P*)	0.679	0.009	0.015	0.208	0.699	

## Discussion

BMD in the THA perioperative period is an issue frequently discussed in the literature. The patients included in our study had a mean BMD value of 0.98 (SD=0.18), measured with DXA. The results of our study suggest that BMD significantly positively correlates with the intraoperatively measured diameter of the femoral neck in the line of neck resection (*r *= 0.5, *P *= 0.009) and with the measured thickness of the cortical bone in the line of neck resection (*r *= 0.47, *P *= 0.015). Furthermore, the CI significantly positively correlates with the intraoperatively measured diameter of the femoral neck in the line of neck resection (*r *= 0.6, *P *= 0.001). We also noted a significant positive correlation between the intraoperatively measured diameter of the femoral neck in the line of neck resection and the intraoperatively measured thickness of the cortical bone in the line of neck resection (*r *= 0.59, *P *= 0.001) as well as a significant positive correlation between CI and CC ratio (*r *= 0.44, *P *= 0.024). All of the other correlations between the age of the patients, CC ratio, Cl, BMD, intraoperatively measured diameter of the femoral neck in the line of neck resection, and intraoperatively measured thickness of the cortical bone in the line of neck resection were not statistically significant.

The authors proposed and examined several methods of measuring the BMD of the patients.

The technique utilized in our study is DXA. The method measures the quantity of X-ray beam that is attenuated by the examined bone region. DXA has several advantages such as a short time of examination, minor patients’ exposure to radiation, broad availability, and affordability [13,14]. There are even some studies suggesting to include the DXA as a routine examination before THA [15]. On the other hand, the examination can be distorted by artifacts, and it is unable to differentiate the bone cortex from the cancellous bone [13,14]. Nevertheless, to our knowledge, the DXA is one of the most commonly used techniques to measure BMD in studies in a similar interest area [15-18].

BMD can also be assessed on CT scans. The authors of the studies employing this method describe its major advantage which is the ability to examine the BMD three-dimensionally. The technique allows for insights into the spatial bone quality and its assessment. Although the CT examination comes with considerable radiation exposure. Even a low-dose CT, utilized in some studies, is not deprived of this disadvantage [14,19,20].

Some authors evaluate other methods in BMD assessment. Klotz et al. compared the trabecular torque measurements with DXA and CT measurements in the study conducted on frozen human femurs. The authors claimed that the method utilizing torque sensors is safe and that it can provide valuable data about bone quality. Nevertheless, they concluded that the technique needs further examination before its clinical use [21].

The timing of BMD measurement varies based on the study's design and protocol. We decided to measure the patients preoperatively. This way, we will be able to observe the changes in the BMD and compare the pre- to postoperative values in further research. We were not the only authors who have chosen to conduct the study with the preoperative BMD measurement before THA [15,16,19,20]. However, in some studies, the measurement was made only postoperatively [17,18]. This protocol gives useful insights into the postoperative changes in the BMD, although it deprives the reader of the context that would be given if the postoperative data were supported by preoperative values.

When it comes to comparing our results with the other studies concentrated on measuring the BMD in the THA perioperative period, we can point out some differences and similarities. In their prospective study, Sariali et al. reported that the preoperatively measured BMD can be a predictor for the short-term patient-reported results [20]. In this phase, our study was not concentrated on the patient-reported outcomes, so we cannot make a definite statement in this matter. For now, we cannot confirm if the findings of our study correlate with the patient’s satisfaction because we have not gathered such information. Examination of this relation should be the point of the following studies. In the retrospective study conducted by Delsmann et al., the authors suggested that DXA measurements ought to be included in a routine procedure before THA. The statement was supported by the results. Among 268 elderly (>70 years) patients, 49 were diagnosed with osteoporosis [15]. In our study, we have not noted any patients with osteoporosis. This may be due to the small sample size. Another reason for the discrepancy may be that the age of the participants of the previously mentioned study was above 70 years, so osteoporosis was more common, whereas in our study, we included younger patients. Other studies with a younger study group seemed to report a lower percentage of patients with osteoporosis. Mühlenfeld et al. in their retrospective study examined 25 patients who underwent total knee arthroplasty (TKA) and another 25 patients who underwent THA. The author measured the patient’s BMD with DXA. The results state that despite the younger age the rate of osteoporosis was higher in the TKA group than in the THA group (32% vs. 12%) [16].

Our study has several advantages when compared to the literature concentrated on similar topics. First, the patients for our study were recruited prospectively and were examined with the DXA before the operation, which will allow for further observation and comparison in the future. Second, the study is based on the DXA measurements, a technique that is safe, affordable, widely available, and commonly used to assess the BMD. Consequently, the results can be easily compared to those of other studies. Next, in the study, we utilized the CI and CC ratio, which allows us to assess the relation between the cortex and the cancellous bone contrary to DXA, which cannot differentiate the cortex from the cancellous bone.

In the matter of the limitations of our study, we recognize some points. First, the size of the study group could be larger. However, when comparing it to recently published studies from a similar interest area, there is no discrepancy in the number of examined patients [16,19]. Second, the measurements with the DXA technique did not allow for the assessment of the bone quality three-dimensionally. That is a disadvantage when comparing our study to the ones utilizing CT in measuring BMD. However, we did not want to compromise the patient’s health by exposing them to the radiation that is exceedingly higher in the CT than in the DXA method. Finally, the surgeries in our study were performed by the same surgeon with the same technique. Nevertheless, the angle of cut in the femoral neck resection might have been slightly different between the patients which could have affected the measurement of the thickness of cortical bone. Perhaps a study utilizing robotic-assisted THA would be more accurate when it comes to a standardized angle of cut in the femoral neck resection.

## Conclusions

BMD measurements can be used in THA preoperative planning to assess bone quality, as they significantly and positively correlate with intraoperative measurements, including the diameter of the femoral neck and the measured thickness of the cortical bone in the line of neck resection. CI significantly and positively correlates with the intraoperatively measured diameter of the femoral neck. However, it does not exhibit a significant correlation with the intraoperatively measured thickness of the cortical bone in the line of neck resection. The CC ratio does not significantly correlate with either the intraoperatively measured diameter of the femoral neck or the intraoperatively measured thickness of the cortical bone in the line of neck resection.
